# Research and development of a new RF-assisted device for bloodless rapid transection of the liver: Computational modeling and in vivo experiments

**DOI:** 10.1186/1475-925X-8-6

**Published:** 2009-03-18

**Authors:** Fernando Burdío, Enrique J Berjano, Ana Navarro, José M Burdío, Luis Grande, Ana Gonzalez, Ignacio Cruz, Antonio Güemes, Ramón Sousa, Jorge Subirá, Tomás Castiella, Ignasi Poves, Juan L Lequerica

**Affiliations:** 1 Department of Surgery, Hospital del Mar, Barcelona, Spain; 2 Instituto de Investigación e Innovación e Bioingeniería (I3BH), Universitat Politècnica de València, València, Spain; 3 Department of Surgery A, Hospital Clínico Universitario Lozano Blesa, Zaragoza, Spain; 4 Department of Electric Engineering and Communications, University of Zaragoza, Zaragoza, Spain; 5 Department of Animal Pathology and Surgery, Veterinary Faculty, University of Zaragoza, Zaragoza, Spain; 6 Department of Urology, Hospital Clínico Universitario Lozano Blesa, Zaragoza, Spain; 7 Department of Pathology, Hospital Clínico Universitario Lozano Blesa, Zaragoza, Spain; 8 Cardiac Research Laboratory, Instituto de Biomedicina de Valencia, Spanish National Research Council (CSIC), Valencia, Spain

## Abstract

**Background:**

Efficient and safe transection of biological tissue in liver surgery is strongly dependent on the ability to address both parenchymal division and hemostasis simultaneously. In addition to the conventional clamp crushing or finger fracture methods other techniques based on radiofrequency (RF) currents have been extensively employed to reduce intraoperative blood loss. In this paper we present our broad research plan for a new RF-assisted device for bloodless, rapid resection of the liver.

**Methods:**

Our research plan includes computer modeling and in vivo studies. Computer modeling was based on the Finite Element Method (FEM) and allowed us to estimate the distribution of electrical power deposited in the tissue, along with assessing the effect of the characteristics of the device on the temperature profiles. Studies based on in vivo pig liver models provided a comparison of the performance of the new device with other techniques (saline-linked technology) currently employed in clinical practice. Finally, the plan includes a pilot clinical trial, in which both the new device and the accessory equipment are seen to comply with all safety requirements.

**Results:**

The FEM results showed a high electrical gradient around the tip of the blade, responsible for the maximal increase of temperature at that point, where temperature reached 100°C in only 3.85 s. Other hot points with lower temperatures were located at the proximal edge of the device. Additional simulations with an electrically insulated blade produced more uniform and larger lesions (assessed as the 55°C isotherm) than the electrically conducting blade. The in vivo study, in turn, showed greater transection speed (3 ± 0 and 3 ± 1 cm^2^/min for the new device in the open and laparoscopic approaches respectively) and also lower blood loss (70 ± 74 and 26 ± 34 mL) during transection of the liver, as compared to saline-linked technology (2 ± 1 cm^2^/min with P = 0.002, and 527 ± 273 mL with P = 0.001).

**Conclusion:**

A new RF-assisted device for bloodless, rapid liver resection was designed, built and tested. The results demonstrate the potential advantages of this device over others currently employed.

## Background

The history of the development of surgical liver resection techniques is largely that of the struggle against hemorrhaging. Before the 1980s, hepatic resection was associated with a mortality rate of 10–20%, mostly due to hemorrhage during liver transection. Nowadays, the hospital mortality rate of liver resection is 5% or lower in most specialized centers [1]. Intraoperative blood loss and perioperative transfusion not only increase the risk of operative morbidity and mortality [2] but also jeopardize long-term survival, since they actually increase the recurrence rate of the tumor being resected [3-5]. As a result, many techniques have been developed over the last ten years to minimize intraoperative blood loss during this type of operation. In addition to the conventional finger fracture, other techniques such as the ultrasonic dissector [6], water jet dissector [7,8], argon beam coagulator [9] and saline-linked radiofrequency (RF) technology [10,11] have been intensively employed to reduce intraoperative blood loss.

Several recent approaches, including saline-linked technology, employ RF energy deposited in the liver tissue to achieve a coagulating effect prior to transection. Weber et al [12] pioneered the use of RF needle electrodes to obtain a 1 or 2 cm wide coagulation band on the resection line before employing the scalpel, thereby facilitating bloodless liver resection. Further applications of the concept of pre-coagulating tissue with RF energy prior to transection have recently been developed with devices like Habib4x [13] or InLine [14-18], in which a range of RF electrodes are inserted in a monopolar or bipolar configuration. Once the plane of coagulation has been created with these RF-assisted devices, the coagulated tissue is cut bloodlessly with a straight scalpel.

The aim of the present study is to present our mutidisciplinary research, including both bioengineering and experimental approaches, to test the efficiency and safety of the new RF-assisted device.

## Methods

### Description of the RF-assisted device

**Figure 1:**
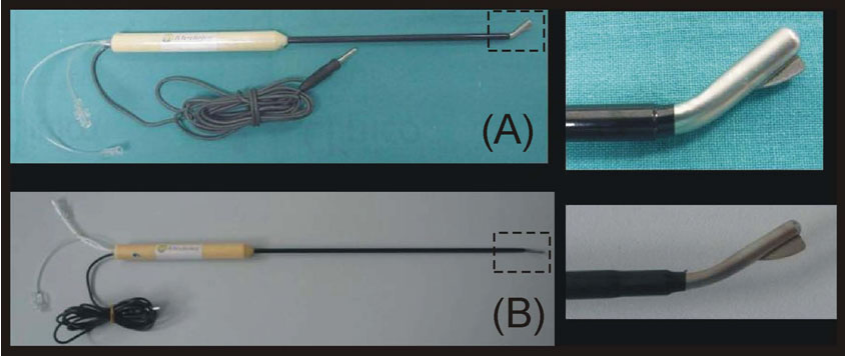
**The new RF-assisted device for bloodless rapid resection in open (A) and laparoscopic approach (B)**. Photos on the right show details of the non-insulated tip (5 and 3 mm in outer diameter, for open and laparoscopic approach, respectively). Note the small sharp blade attached to the very tip of the instrument and the blunt bladeless side of the tip.

To date, the RF-assisted device has been manufactured by Minimeca-Medelec (Puidoux, Switzertland) as a prototype. The device and its operating procedure have been described previously for both open [19] and laparoscopic approaches [20]. Briefly, it consists of a handheld instrument that conducts two surgical tasks simultaneously: coagulation and cutting. A photograph of the devices is shown in Figure 1, which shows the open approach model (A) and the laparoscopic (B) (only the dimensions are different). The coagulation task is performed by a blunt-tip metallic electrode connected to an CC-1 Cosman Coagulator System (Radionics, Burlington, MA, USA) operating at maximum power (≈90 W) in manual mode. The electrode is comprised of two internal closed lumens, one of which delivers chilled saline (0°C) to the distal tip by means of a peristaltic pump (Radionics, Burlington, MA, USA) at a rate of approximately 130 mL/min and the other returns the warmed solution to an external collection assembly. The cutting task is carried out by a sharp 2 mm long blade attached distally to the tip (see Figure 1).

**Figure 2:**
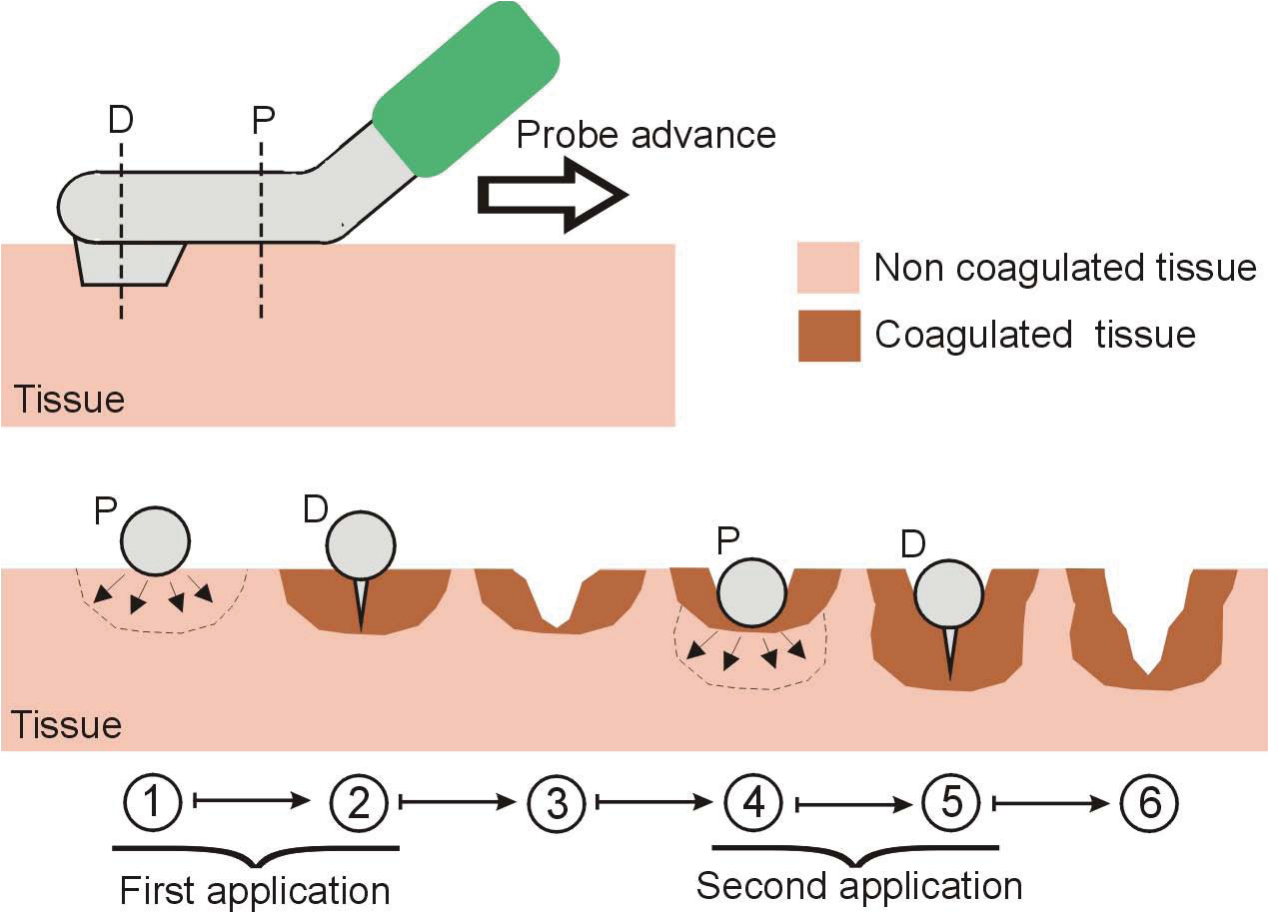
**Main function of the new RF-assisted device for rapid bloodless resection**. Top: Lateral view of the probe showing the distal section (D) with the blade, the proximal section (P) joined to the insulated part of the probe (green), and the advance direction of the probe on the target tissue. Bottom: Cross-sectional views of a fragment of target tissue showing two sequential applications. Each application consists of two steps: first, the tissue is heated (and coagulated) by applying radiofrequency currents (arrows) using the proximal section (steps 1 and 4), after which the blade of the distal section resects the previously coagulated tissue (steps 2 and 5) [18].

The main function of the new device is to cut pre-coagulated tissue. Figure 2 shows cross-sectional views of a fragment of target tissue at different stages. Briefly, the surgeon moves the device back superficially touching the tissue to be transected with the distal side of the device. The blunt proximal tip coagulates the tissue with a backward movement and this is subsequently transected by the blade located distally at the tip. The sharp blade only cuts the tissue that has previously been coagulated (generally 2 mm) and provides new coagulated tissue for the next pass with the instrument. The tissue is therefore homogeneously coagulated only once (and is not overheated) due to the homogeneous cooling effect provided by the internal closed-circuit saline infusion at the tip. The tissue does not stick to the instrument and a homogeneous depth of coagulated tissue can be expected, whatever the angle between the tissue and the tip. The pre-coagulated tissue is then cut bloodlessly to a precise depth with the blade attached to the very tip of the instrument. In contrast to conventional saline-linked devices, which provide a continuous stream of saline onto the tissue, no pooling of saline at the transection plane is observed with the proposed device. Diffusion of the electric current is therefore not impaired by an excess or scarcity of saline on the transection plane and homogenous contact is maintained with the tissue, reducing the need to use the sucker and improving visualization of the transection plane.

**Figure 3:**
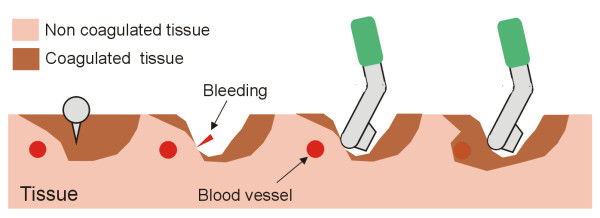
**Secondary function of the new RF-assisted device for rapid bloodless resection**. Cross-sectional views of a fragment of target tissue showing a nearby blood vessel which impedes full coagulation. This secondary function may be employed whenever a bleeding point appears. The surgeon moves the bladeless side of the instrument continuously over the tissue in circular passes close to the bleeding point in order to achieve hemostasis.

The additional function of the new device is surface coagulation without cutting in order to achieve hemostasis. The surgeon moves the bladeless side of the instrument continuously over the tissue to be coagulated in circular passes until hemostasis is achieved (see Figure 3).

### Computer modeling

Computer modeling is widely employed to study the electrical-thermal performance of electrodes and applicators in RF heating of biological tissues [21]. Briefly, the temperature (*T*) in the tissue is obtained by solving the *Bioheat Equation*, which governs thermal phenomena during RF heating of biological tissues:

(1) $$\rho \cdot c\cdot \frac{\partial T}{\partial t}=\nabla \cdot (k\nabla T)+q-Q_{p}$$

where *ρ*, *c *and *k *are respectively the density, specific heat and thermal conductivity of the tissue. The term *Q*_*p *_corresponds with the heat loss caused by blood perfusion. Our study modeled an ex vivo transection, and hence we did not consider this term. Finally, the term *q *is the heat source caused by RF power (Joule loss) which is given by:

(2) *q *= *J*·*E*

where *J *is the current density (A/m^2^) and *E *is the electric field intensity (V/m). The values of these two vectors are evaluated using Laplace's equation:

(3) ∇·*σ*∇Φ = 0

where Φ is the voltage (V) and *σ *is the electrical conductivity (S/m).

**Figure 4:**
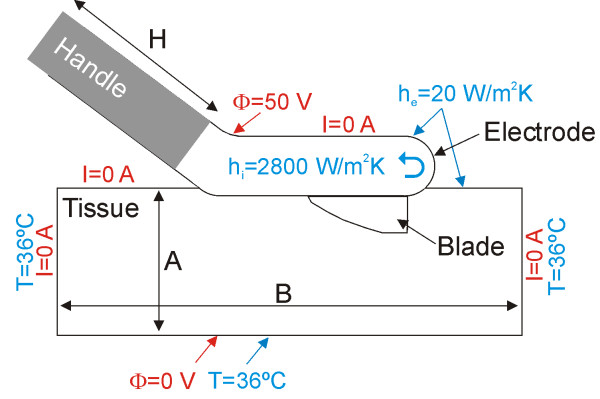
**Theoretical model considered in the computer modeling study (out of scale)**. The sketch only shows the symmetry plane cutting the applicator (handle + electrode + blade). Physical dimensions of the applicator correspond with those of the real device, while the tissue dimensions (depth is not shown but is identical to A) were calculated by means of a convergence analysis. Thermal (blue) and electrical (red) boundary conditions are also shown.

**Figure 5:**
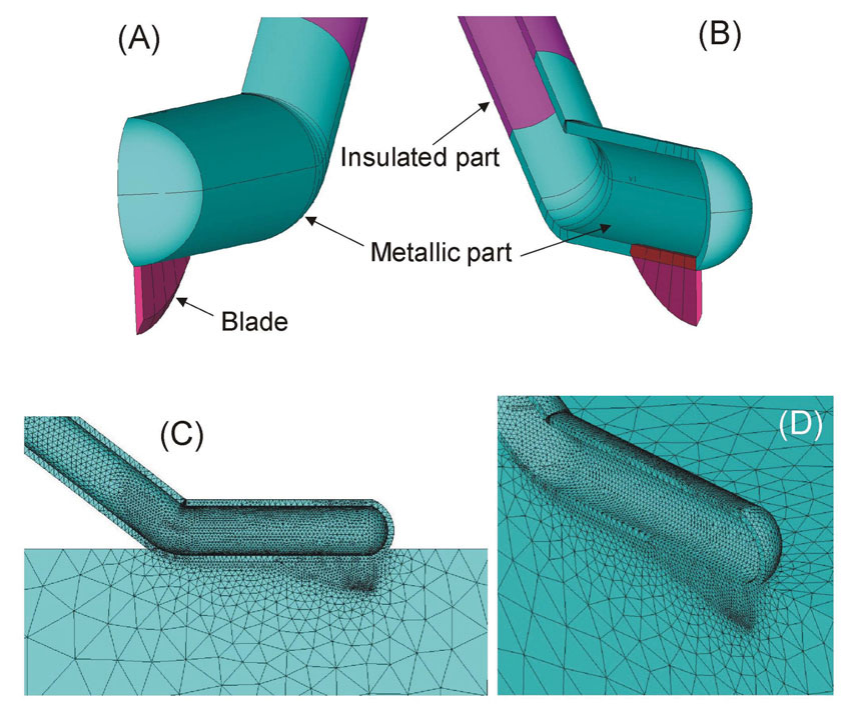
**A and B are two views of the computational model of the new RF-assisted device for rapid bloodless resection**. The model had a symmetry plane, only half of the electrode and tissue was considered. The insulated part corresponds to the plastic holder. Inner cooling was not modeled realistically by internal tubes, but only by the outer metallic part, together with a thermal boundary condition of convective coefficient (h_i_). C and D are two views of the Finite Element Model built.

Table 1 shows the thermal and electrical characteristics of the model elements. We used the ANSYS program (ANSYS, Canonsburg, PA, USA) for the creation of Finite Element Models and for solving the above equations by computer simulations [21]. Regarding surgical devices for RF-assisted transection, computer modeling studies have been proposed to study the current density distributions in the tissue, and therefore also the subsequent temperature distributions [22]. For our research, we built a theoretical model of the RF-assisted device, as shown photo (A) of the Figure 1, with the device in contact with a fragment of hepatic tissue (see Figure 4). We considered an insertion depth of 1 mm between device and tissue. The model had a symmetry plane, and hence we only considered half of the electrode and tissue fragment. Figure (5A and 5B) shows a detail of the device modeled, which was considered to be empty. Internal cooling was not modeled realistically by means of internal tubes, but we considered only the metallic outer part, and then used a thermal boundary condition with a convective coefficient (*h*_*i*_) to model the cooling effect of the circulating fluid. The parameter *h*_*i *_was estimated by using the theoretical calculation of forced convection inside a tube of diameter 4 mm [23]. The value of *h*_*i *_for laminar flow was calculated using:

**Table 1:** Characteristics of the elements employed in the computer modeling.

**Element**	***σ*** **(S/m)**	***ρ*** **(kg/m3)**	***c*** **(J/kg·K)**	***k*** **(W/m·K)**
Electrode and conducting blade	7.4 × 10^6^	8 × 10^3^	480	15
Insulated handled and insulated blade	10^-10^	2200	1050	0.35
Hepatic tissue	0.33*	1060	3600	0.5

*σ*: electrical conductivity; *ρ*: mass density; *c*: specific heat; and *k*: thermal conductivity.

* Assessed at 36°C. The change of *σ *with temperature was of +1.6/°C.

(4) $$Nu=\frac{h_{i}\cdot L}{k_{f}}$$

where *Nu *is the Nusselt number (dimensionless), *k*_*f *_is the thermal conductivity of circulating fluid (W/m·K), *L *is the length of the heated area (parallel to the direction of the flow), which assumed to be 15 mm. The average Nusselt number ($\bar{Nu}$) for laminar flow, constant heat flux from a plate heated along length *L*, can be estimated from the equation:

(5) Nu¯=0.664⋅(Re⁡1/2)⋅(Pr⁡1/3)

where Re is the Reynolds number and Pr is the Prandtl number (both dimensionless). The equation (5) is valid for Re < 5 × 10^5^. These numbers are calculated from the equations:

(6) Re⁡=ρf⋅u⋅Lμ

(7) $$\mathrm{Pr}=\frac{c_{f}\cdot \mu }{k_{f}}$$

where *ρ*_*f *_is the density of circulating fluid (Kg/m^3^), *μ *is the dynamic viscosity (N·s/m), *c*_*f *_is the specific heat at constant pressure (J/kg·K) and *u *is the velocity of the flow (m/s) calculated as:

(8) $$u=\frac{F}{60\times 1000\times A_{t}}$$

where *F *is the flow of the circulating fluid (0.13 L/min) and *A*_*t *_is the cross-sectional area of the tube = *π*·(0.002)^2 ^= 1.2566 × 10^-5 ^m^2^. Flow velocity was therefore ≈0.172 m/s.

The characteristics of the circulating fluid were considered to be those of water at 37°C [23]: *k*_*f *_= 0.63 W/m·K, *ρ*_*f *_= 999.4 Kg/m^3^, *μ *= 6.9 × 10^-4 ^N·s/m, and *c*_*f *_= 4174 J/kg·K. We obtained a Reynolds number Re = 3737, and a Prandtl number of Pr = 4.57. As a result, $\bar{Nu}$ ≈ 67, and *h*_*i *_≈ 2800 W/m^2^K. The coolant temperature was considered to be 5°C.

As in every FEM problem, we had to define the boundary conditions. Figure 4 shows the thermal (blue) and electrical (red) boundary conditions. The temperature for surfaces at a distance from the device was assumed to be 36°C (this was also the value for initial temperature). A thermal condition of null thermal flux was used at the symmetry plane. The effect of free heat convection in the tissue-ambient and device-ambient interfaces was taken into account using a thermal transfer coefficient (*h*_*e*_) of value 20 W/m^2^K. A value of 21°C was considered for the ambient temperature.

Regarding the electrical boundary conditions, computer simulations were conducted using a constant electrical voltage of 50 V between the metallic part of the device and dispersive electrode, which was assumed to be on the bottom surface (see Figure 4). The electrical voltage on the dispersive electrode was fixed at zero volts. An electrical condition of null electrical current was used at the symmetry plane. The same condition was used on the surfaces at a distance from the device (except for the bottom surface), and on the tissue-ambient and device-ambient interfaces.

Physical dimensions of the applicator correspond with those of the actual device. However, the tissue dimensions (A and B in Figure 4) and handle length (H in Figure 4) were calculated by means of a sensitivity analysis in order to avoid boundary effects. A convergence test was performed to obtain the adequate spatial and temporal resolution [24]. The value of the maximal temperature achieved in the hepatic tissue (T_max_) after 1 s of heating was used as a control parameter in these sensitivity and convergence tests. The spatial resolution was heterogeneous. We used the following criterion: the finest zone was always the blade-tissue interface because it is known that this has the largest voltage gradient and hence the maximum value of current density. In the tissue, grid size was increased gradually with distance from the blade-tissue interface. First, we considered a tentative spatial and temporal resolution. We then conducted a computer analysis to determine the appropriate values of A, B and H (see Figure 4). These simulations were made by increasing the value of the three parameters by equal amounts. When the difference between the T_max _and the T_max _in the previous simulation was less than 0.5°C, we considered the former values to be adequate. Finally, we performed convergence tests to determine adequate spatial and temporal resolution. The spatial resolution was achieved by refining the mesh near the blade so that T_max _was within 0.5% of the value obtained from the previous refinement step [24]. With an adequate spatial resolution achieved, we decreased the time step until T_max _was within 0.5% of the value obtained from the last time step.

In this study, the simulations were stopped when maximal temperature in the tissue reached 100°C [25]. This was because no experimental data have been reported dealing with the thermal and electrical characteristics of hepatic tissue above this temperature. We then analyzed the voltage and temperature distributions in the tissue using the 55°C isotherm as thermal lesion boundary. The model was employed to assess the electrical-thermal performance of the proposed device, i.e. to determine the location of the hottest points. It should be pointed out that at the hottest points, i.e. in the tissue zones where temperature reaches 100°C, dehydration and carbonization processes occur. These phenomena can drastically limit the progress of the lesion, and hence the expansion of the coagulated area. Furthermore, carbonization can cause carbonized tissue to adhere to the electrode, which could affect the mechanical characteristics of some of its critical parts, such as the blade. In fact, in view of the results of the first simulation, we modified the model to study the effect on the electrical characteristics of the blade. To be more precise, we performed a further computer simulation considering a blade made of the same non conducting material as the handle (see Table 1).

### In vivo study on pig liver

Sixteen partial hepatectomies were performed on pig liver with the proposed device by both laparotomy (n = 8) and a laparoscopic hand-assisted approach (n = 8). Eight similar partial hepatectomies were also performed using a conventional saline-linked device through laparotomy (DS 3.0; Tissue-Link Medical, Dover, New Hampshire, USA) acting as a control group (used in accordance with the manufacturer's recommendations). A total of 12 female farm pigs were employed in the study. The animals were deeply anesthetized with Tiletamine-Zolazepam (7 mg/kg, im), medetomidine (0.03 mg/kg) and maintained with propofol (10 mg/kg) or sevoflurane. In all cases, the specific efficiency of each medical device (proposed and the Tissue-link^® ^device) was evaluated in division and hemostasis without the aid of any other instruments unless a hemorrhage of more than 2 min was observed. After performing the experiment the animals were euthanized by exanguination. All the experimental procedures were conducted in a laboratory authorized for animal research and approved by the local institutional ethical committee.

The main outcome measures of the study were: 1) *Transection time*: total time of transection including time for achieving complete hemostasis; 2) *Blood loss*: total amount of blood loss during transection (from the sucker and bloody gauzes minus saline dripped onto the tissue); 3) *Transection area*: obtained by delineation of the transection plane (digital photograph) using appropriate software (3D Doctor, Able Software Corp, Lexington, MA, USA); 4) *Transection speed*: ratio of the transection area to transection time, 5) *Blood loss per transection *area and 6) Tissue *coagulation depth*: mean tissue coagulation depth calculated by histological assessment from four points equidistant from the mid transverse line of the transection area in every case. The hemorrhagic rim surrounding ablation tissue was not included in coagulation depth measurements.

The statistical analyses were performed on mean values in the open approach (proposed device vs. Tissue-link^® ^method). Mean values of numerical data were compared for both groups using the Student *t *test or the U-Mann-Whitney's test when appropriate. Categorical data were compared for both groups using Fisher's exact text. Differences in variables were considered to be significant at a threshold of *P *< 0.05. Statistical analyses were performed with statistical software (version 12.0; SPSS, Chicago, IL, USA).

### Technical modifications for clinical trials

**Figure 6:**
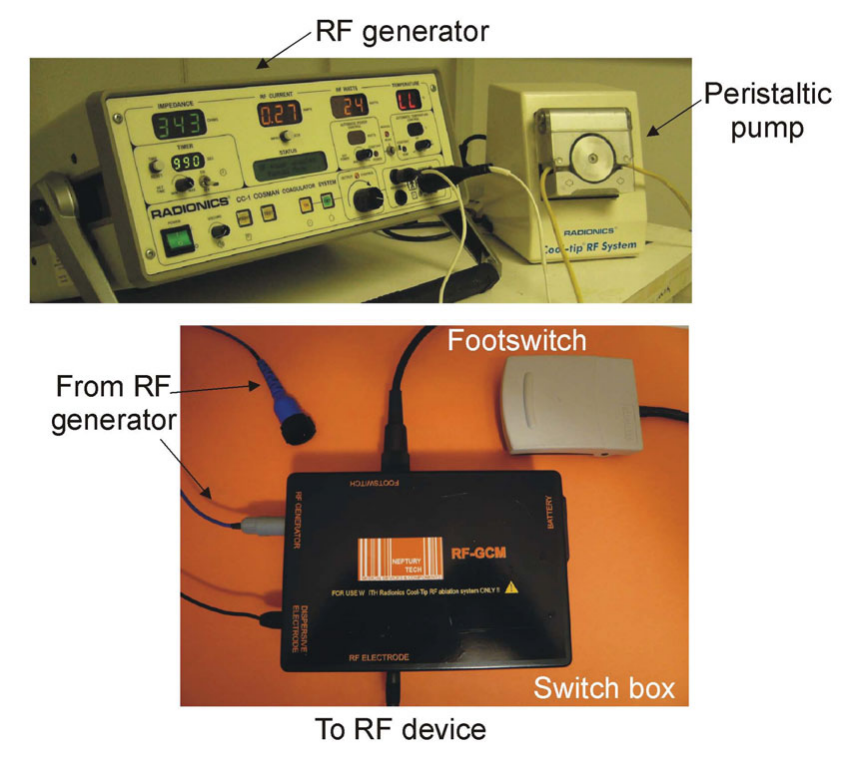
**Top: Radiofrequency (RF) generator and peristaltic pump employed respectively to deliver RF currents and to cool the new device**. Bottom: Switch bow designed to serve as interface between the device and the RF generator. RF power is controlled via a footswitch.

Certain technical modifications had to be made to the instrumentation employed for the clinical trials. To date, the RF generator used in all in vivo experiments had been the CC-1 Cosman Coagulator System RF generator (Radionics, Burlington, MA, USA) (see Figure 6). However, this RF generator was designed for RF ablation of tumors by percutaneous electrodes inserted into the target tumor. During RF power delivery, tissue impedance remains approximately inside a certain range. In fact, when tissue impedance goes over a pre-set value (200–300 Ω) the RF generator switches off, and it also has a lower impedance limit. On the other hand, when this generator is connected to a surgical-oriented device such as the Tissue-Link^®^, sudden large changes in tissue impedance may occur. In fact, during an operation, the surgeon may remove the device from the tissue and thus create an open circuit (i.e. very high impedance), which would make the generator switch off. Furthermore, the proposed device for RF-assisted resection should be controlled by a foot switch (operated by the surgeon) rather than by a push-button on the front panel of the RF generator (typical arrangement for RF ablation). To solve these problems, we designed a switchbox (Neptury Technologies, Almassora, Spain) which acts as interface between the RF generator and the device (Figure 6), based on a relay controlled by a foot-switch MKF 1S-MED (Steute Medizintechnik, Löhne, Germany). The assistant programs a power value on the generator front panel and from then on RF power control is exclusively in the hands of the surgeon. On the other hand, in order to avoid problems with the tissue impedance range, the switchbox includes internal power resistors which balance the total impedance measured by the RF generator, even under extreme conditions (short and open circuit). Finally, in order to comply with regulations, the switchbox is powered by internal batteries.

## Results

### Computer modeling

After the sensitivity analyses, we obtained the following dimensions: A = 30 mm, B = 45 mm and H = 10 mm. The convergence test provided a grid size of 0.1 mm in the finest zone (blade-tissue interface), and a step time of 0.05 s. The model had nearly 14,800 nodes and used over 66,000 tetrahedral elements (see C and D in Figure 5).

**Figure 7:**
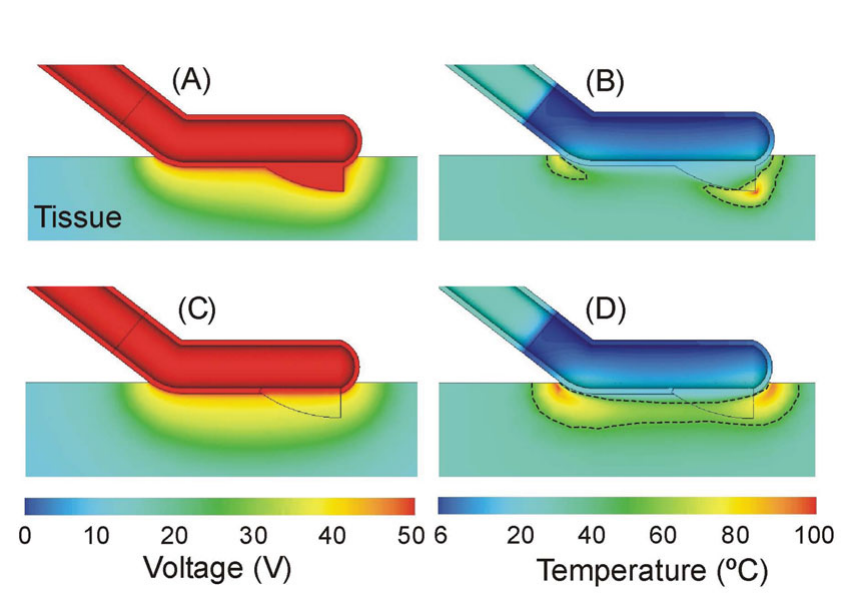
**Results of the computer simulations**. Voltage (A and C) and temperature (B and D) distributions in the tissue. Temperature distributions correspond to the time when maximal tissue temperature reaches 100°C. Figures A and B show the case of a metal blade in electrical contact with the electrode body (t = 3.85 s), while Figures C and D show the case of an insulated (non-conducting) blade (t = 10 s). The voltage applied was of 50 V in both cases. Dashed line represents 55°C isotherm and serves as lesion boundary marker.

Figure 7 shows the voltage and temperature distributions in the tissue for the two computational models: metal blade (A and B) and insulated blade (C and D). Regarding the electrical performance of the conventional device (i.e. metal blade), plot A in Figure 7 shows a high electrical gradient around the tip of the blade. This produces considerable Joule heating at this point, which results in the maximal increase of temperature, as can be observed in Figure 7(B). A temperature of 100°C was reached in 3.85 s. Other hot points with lower temperatures were located at the proximal edge of the device.

A comparison of the results provided by the two blades showed that the electrically insulated blade took longer to reach 100°C tissue temperature (10 s) than the electrically conducting blade (3.85 s). As a result, the lesion boundary (assessed as the 55°C isotherm) was more uniform and larger in the case of the electrically insulated blade (see D in Figure 7).

### Performance of the proposed device on in vivo pig liver

**Figure 8:**
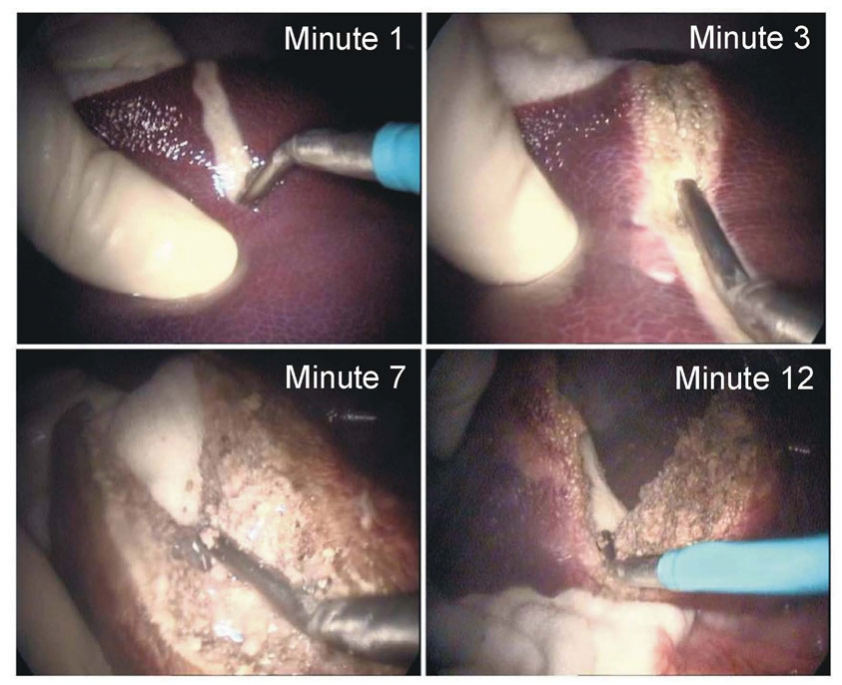
**Four steps (from left to right and top to bottom) during transection of the liver with a hand-assisted laparoscopic approach with the proposed device (total time: 13 minutes)**. See text for further details. Note that no other device is used to achieve dissection, parenchyma division and hemostasis.

All the animals tolerated the procedures well, and no significant complications occurred during the operative procedure. During transection, one or two vein vessels (often more than 5 mm in diameter) were encountered. With conventional saline-linked technology, in almost all cases (7/8) these large vessels required one or two stitches to achieve complete hemostasis after 2 min of continuous bleeding, even though the saline-linked device was used intensively, following the manufacturer's recommendations. Conversely, with the proposed method, no other instrument was employed during liver transection in any case, either in the open or the laparoscopic approach (Figure 8, Additional file 1). As shown in Table 2, the proposed RF-assisted device achieved a 30% increase in mean transection speed as compared to the saline-linked technology in similar conditions. Even more important, mean blood loss per transection area was nearly seven times lower with the proposed method than the saline-linked method (Table 2). Using the proposed method in the laparoscopic approach, similar or even better figures were obtained than with the open approach with the same device both in transection speed and blood loss per transection area.

**Table 2:** Results of the in vivo studies comparing the new device with Tissue-Link^®^.

	**New device under test**		**Tissue-Link^®^**	P*
**Output variable**	Open	Laparoscopic	Open	**-**

Transection time (min)	12 ± 3	13 ± 7	21 ± 7	**0.006**
Blood loss (mL)	70 ± 74	26 ± 34	527 ± 273	**0.001**
Transection area (cm^2^)	35 ± 7	34 ± 11	41 ± 8	N.S.
Transection speed (cm^2^/min)	3 ± 0	3 ± 1	2 ± 1	**0.002**
Blood loss *per *transection area (mL/cm^2^)	2 ± 2	1 ± 1	13 ± 6	**0.001**
Tissue coagulation depth (mm)	6 ± 2	9 ± 2	3 ± 1	**0.005**

* Mean values were compared between both methods only in the open approach.

Differences in variables were considered to be significant at a threshold of *P *< 0.05.

N.S: no significant differences.

## Discussion

This paper describes the different steps followed by our group in the R&D of a new RF-assisted device for rapid, bloodless liver resection. We first employed the computer modeling technique to predict and assess the temperature profile of the new device. This approach has been widely employed in surgical research, not only in RF heating of biological tissue [21], but also to assist the design of endoscopic instruments [26], to assess the impact of surgical procedures [27] and to evaluate the performance of different surgical devices [28]. The advantages of this approach are its low cost and the short time needed to determine basic performance and the effect of different parameters (e.g. dispersion in the geometry and physical characteristics of the tissues, materials and designs of surgical devices). The main disadvantage is obviously the lack of accurate characterization of the biological tissue (i.e. full data on thermal, electrical and mechanical properties). In our study, the results of the computer modeling showed a marked edge effect at the blade tip (see B in Figure 7), which has also been observed in long electrodes in surface application for cardiac ablation [29]. This overheating at the tip could produce rapid tissue carbonization adhering to the blade, and hence could impair the cutting quality. For this reason, we conducted additional simulations with a non (electrically) conducting blade and the results showed a considerable reduction of the edge effect. Maximal temperature was not located at the blade tip, avoiding the risk of carbonization in this zone, and hence preserving cutting quality. In view of these results, future studies should be conducted using prototypes with electrically insulated blades. However, it may be difficult to find a high-performance non electrically conducting material appropriate for surgical blades.

Regarding the research on a surgical device for bloodless hepatic resection, it is very important to point out that ex vivo setups do not allow modeling of blood perfusion, and it will therefore be necessary to plan further in vivo experiments. We did, in fact, conduct in vivo experiments on the pig model to assess the true potential of the new device [19,20], as other authors had done previously [22,30]. In addition, our in vivo studies were always planned as comparative assessments. We paid particular attention to comparing our device with the Tissue-Link^® ^device (DS 3.0; Tissue Link Medical, Dover, New Hampshire, USA). This is a conventional saline-linked system which acted as the control group in our experiments. It employs similar technology to the proposed device and shows promise but is still under evaluation [11,31]. The performance of our device was demonstrated in the open comparative approach, especially in the 30% increase in liver transection speed (over the Tissue-Link^® ^device) (see Table 2). Even more important, our device demonstrated a seven-fold reduction in blood loss during liver transection (over the Tissue-Link^® ^device) (see Table 2). With the proposed method a greater mean coagulation depth was obtained compared to the saline-linked device (6 ± 2 mm and 3 ± 1 mm, for the new device and Tissue-link device, respectively). This was the only macroscopic or microscopic difference found between transection surfaces in both groups.

The laparoscopic study with our device on the other hand, demonstrated similar or even better results than in the open approach with our device [20]. Hence, our device may be used with a minimally invasive approach in a performance fashion.

### Clinical trials

Even though the device gave good results in both open and laparoscopic approaches in the tests on animals, further clinical research, especially through a Randomized Controlled Trial -RCT- will be necessary before the system can be introduced into clinical practice [32]. At the moment we are in the process of designing a two-phase clinical testing program. Phase I study will examine the safety of the procedure with a small sample of patients suffering from liver metastases. When and if the safety of the procedure is guaranteed, Phase II study will evaluate the new device in comparison to a conventional method of liver transection with randomly selected patients.

### Limitations of the study

This study presents the following limitations:

1) The method studied has been compared with the saline-linked dissecting sealer, which is a promising technology but is still under evaluation. It will therefore be necessary for future research by other groups to compare the studied method with other available technologies, such as CUSA or Kelly crashing.

2) The method sacrifices parenchymal tissue that is usually spared in other resectional techniques. This could be especially relevant in cirrhotic patients with limited remnant reserve.

3) In the study of a pig liver model, we tested the *specific *efficiency of each procedure in division and hemostasis without the aid of other instruments. It is conceivable that in an actual clinical setting (combining these methods with other systems of division and hemostasis when necessary) differences in transection time and transection speed between both methods may be smaller.

## Conclusion

In this paper we have presented our research and development plan for a new RF-assisted device for rapid, bloodless liver resection, covering a variety of different techniques ranging from computer modeling to clinical trials. The interaction of these multidisciplinary methodologies is expected to provide further data on the new device.

## Competing interests

Drs. FB and AGU have applied for a patent relating to the content of the manuscript. All other authors declare that they have no competing interests.

## Authors' contributions

FB and EJB designed and coordinated the research plan and conducted computer modeling studies; AN, JMB, AGO, IC, AGU, RS and JS and carried out the in vivo experiments; LG participated in the design of the in vivo experiments and helped to draft the manuscript; TC conducted the histopathologic examinations; IP and JLL participated in the design of technological improvements. All authors have read and approved the final manuscript.

## Supplementary Material

Additional file 1 **Transection of the liver with a hand-assisted laparoscopic approach with the proposed device.** Note that no other device is used to achieve dissection, parenchyma division and hemostasis. Click here for file
